# Complementing Tissue Testing With Plasma Mutation Profiling Improves Therapeutic Decision-Making for Patients With Lung Cancer

**DOI:** 10.3389/fmed.2022.758464

**Published:** 2022-02-11

**Authors:** Yukti Choudhury, Min-Han Tan, Jun Li Shi, Augustine Tee, Kao Chin Ngeow, Jonathan Poh, Ruth Rosalyn Goh, Jamie Mong

**Affiliations:** ^1^Lucence Diagnostics Pte. Ltd., Singapore, Singapore; ^2^Lucence Health Inc, Palo Alto, CA, United States; ^3^Institute of Bioengineering and Nanotechnology, Singapore, Singapore; ^4^Department of Respiratory and Critical Care Medicine, Changi General Hospital, Singapore, Singapore; ^5^Faculty of Medicine, Imperial College London, London, United Kingdom; ^6^Institute of Bioengineering and Bioimaging, Singapore, Singapore

**Keywords:** liquid biopsy and circulating tumor DNA, non-small cell lung cancer (NSCLC), plasma-first, next generation sequencing, amplicon-based NGS, tumor heterogeneity, NGS panel testing

## Abstract

**Background:**

Tissue biopsy is an integral part of the diagnostic approach to lung cancer. It is however invasive and limited by heterogeneity. Liquid biopsies may complement tissue testing by providing additional molecular information and may be particularly helpful in patients from whom obtaining sufficient tissue for genomic profiling is challenging.

**Methods:**

Patients with suspected lung cancer (*n* = 71) were prospectively recruited. Blood and diagnostic tissue samples were collected within 48 h of each other. Plasma cell-free DNA (cfDNA) testing was done using an ultrasensitive amplicon-based next-generation sequencing (NGS) panel (plasma NGS testing). For cases diagnosed as non-small cell lung carcinoma (NSCLC) *via* histology or cytology, targeted testing for epidermal growth factor receptor (*EGFR*) mutations was performed using tissue biopsy samples (tissue *EGFR* testing), where available. Concordance of clinically actionable mutations between methods and sample types was assessed.

**Results:**

For confirmed NSCLC cases (*n* = 54), tissue *EGFR* test results were available only for 70.3% (38/54) due to sample inadequacies, compared to blood samples for 98.1% (53/54) cases. Tissue *EGFR* testing identified sensitizing *EGFR* (L858R or exon 19 deletion) mutation in 31.6% (12/38) of cases. Plasma NGS identified clinically actionable mutations in 37.7% (20/53) of cases, including *EGFR* mutations in two cases with no tissue *EGFR* results, and mutations in *KRAS, BRAF*, and *MET*. The overall sensitivity of sensitizing *EGFR* mutation detection by plasma NGS was 75% (9/12), and specificity was 100% (25/25) in patients tested in both tissue *EGFR* and plasma NGS (*n* = 37). In this cohort of patients, tissue *EGFR* testing alone informed clinical decisions in 22.2% (12/54) of cases. Adding plasma NGS to tissue *EGFR* testing increased the detection rate of actionable mutations to 42.6% (23/54), representing a 1.9-fold increase in clinically relevant findings. The average turnaround time of plasma NGS was shorter than standard tissue testing (10 vs. 29.9 days, *p* < 0.05).

**Conclusions:**

In the first-line setting, plasma NGS was highly concordant with tissue *EGFR* testing. Plasma NGS increases the detection of actionable findings with a shorter time to results. This study outlines the clinical utility of complementary plasma mutation profiling in the routine management of lung cancer patients.

## Introduction

Lung cancer is the most common cause of cancer death worldwide and nonsmall cell lung cancer (NSCLC) accounts for 85% of all the lung cancers, making NSCLC a major cause of mortality (1). The 5-year survival rate of lung cancer patients is 18.6% and for late-stage NSCLC the 5-year survival rate stands at 6% (2). The median age of diagnosis of NSCLC is 70 years of age and about 40% of patients are diagnosed with lung cancer at a late stage (2). Given the age profile and time-scarce outlook for the average lung cancer patient, it is important to create diagnostic tools that are fast, sensitive, and accessible by all patients, in particular those of advanced age or cancer stage.

Major progress has been made in the treatment of advanced NSCLC with the identification of specific driver mutations and the development of targeted therapies (3, 4). Although actionable mutations are found in only a subset of patients, progression-free survival was shown to be significantly increased in patients treated with targeted therapy compared to those treated with chemotherapy (5). Molecular diagnostic testing combined with molecular targeted agents directed against driver mutations in *EGFR, ALK, ROS1, BRAF, MET, RET*, and most recently *KRAS* has significantly improved the outcomes for patients with advanced disease harboring these alterations (6, 7). The most recent National Comprehensive Cancer Network (NCCN) guideline recommendations (Version 5.2021) for the management of NSCLC now include testing for *EGFR, BRAF, ALK, ROS1, RET, KRAS, MET* exon 14 skipping, and *NTRK* in nonsquamous lung cancer, as part of broader molecular profiling (8).

Tissue biopsy is the prevailing gold standard for the diagnosis of NSCLC among patients suspected to have lung cancer, and tumor testing is most commonly used for the determination of guideline-recommended biomarkers. In about 15 to 40% of NSCLC cases, comprehensive molecular testing is not feasible due to insufficient tissue samples (9, 10). In the absence of a comprehensive tissue test, a serial testing approach was shown to be successful in only 5% of patients for all the eight guideline-recommended biomarkers (11). Sampling a single lesion may not capture the complete genomic landscape due to molecular heterogeneity of tumors (12). The risk of complications is another concern, rising to 61% with the use of transthoracic needle biopsy, and the incidence of pneumothorax also increases significantly in older patients with obstructive lung disease (13). Another challenge is the time required for guideline-complete tissue testing which can result in a substantial number of patients initiating chemotherapy before diagnostic results become available, with 19% of *EGFR* mutation or ALK rearrangement positive patients initiating first-line chemotherapy while awaiting their biomarker test results (10).

Liquid biopsies present an alternative approach to tissue-based diagnostic testing, with the use of plasma cell-free DNA (cfDNA) as the substrate for molecular profiling. Tumor alterations identified through routine analysis of clinical tissue samples are detected in cfDNA with a sensitivity of ~80–90% (14). Detection sensitivity is influenced by both anatomical sites of disease and tumor burden which in turn correlates with overall circulating tumor DNA (ctDNA) burden (15–17). A recent study focused on the use of cfDNA for the diagnosis of NSCLC found a pooled sensitivity of 68% *via* a systematic review (18). Currently, the NCCN guidelines only endorse (1) a plasma-first approach for testing for EGFR T790M in patients who have developed resistance to first- or second-generation tyrosine kinase inhibitors (TKIs), with tissue biopsy being recommended in cases where plasma testing is negative (8), and (2) liquid biopsy in specific clinical circumstances where the patient is medically unfit for invasive tissue sampling or when tumor tissue specimen is inadequate or unobtainable, following pathological confirmation of diagnosis, with a follow-up tissue-based analysis in cases where no oncogenic driver is identified in plasma cfDNA (8). This is aligned with the latest recommendations from the International Association for the Study of Lung Cancer (IASLC) for liquid biopsy for NSCLC, where liquid biopsy is recommended for cases where the tissue sample is unavailable (“plasma first” approach), or in cases where tissue biopsy is inadequate for conducting comprehensive tissue genotyping (“complementary” approach) (19). Furthermore, according to the IASLC recommendations, for cases with oncogene-addicted NSCLC progressing after initial targeted therapy, a “plasma first” approach should be considered standard of care (19).

Liquid and tissue biopsies each present their own strengths. In this study, we hypothesize that plasma cfDNA testing using a panel of target genes can complement standard molecular testing using tissue biopsy for NSCLC patients. Here, standard molecular tests encompass single target (e.g., *EGFR*) PCR-based tests, which could require time-consuming serial tissue testing depending on previous findings. For plasma cfDNA testing, next-generation sequencing (NGS)-based approaches, if adequately sensitive and comprehensive, have been shown to identify actionable mutations in plasma cfDNA of advanced NSCLC (20, 21). Therefore, rather than substituting tissue biopsies with liquid biopsies, adding a concurrent plasma NGS test to tissue testing would improve the detection of actionable mutations in patients with NSCLC, improving prognostication in addition to choice and timeliness of treatment initiation. This may translate to a “plasma-first” approach where getting a tissue sample is rendered impractical or extremely difficult (19).

This study focuses on standard tissue testing for mutations in the *EGFR* gene, which is mutated in 40–60% of Asian patients and 10–20% of Caucasian patients with NSCLC (22). Specifically, EGFR L858R and in-frame exon 19 deletions account for 50 and 40% of *EGFR* mutations, respectively, and are sensitizing mutations as tumors harboring these mutations are sensitive to EGFR TKIs (23). Molecular testing for alterations in multiple genes such as *EGFR, ALK, ROS1, RET, BRAF, ERRB2, MET* exon 14, and *NTRK1/2/3* have progressively entered the standard of care over the last 10 years (24). Here, we aim to demonstrate the clinical utility of an ultrasensitive, amplicon-based NGS tool for plasma cfDNA testing alongside standard tissue testing in patients suspected to have lung cancer, to widen the scope of eligibility for treatment and reduce waiting time for molecular test results.

## Methods

### Study Design and Patients

Patients with suspected lung cancer (*n* = 71) were prospectively enrolled for this study at the Department of Respiratory Medicine, Changi General Hospital, Singapore between June 2015 and August 2018. Before diagnosis by histology, blood samples for NGS-based plasma genotyping were collected during the patient visit, followed by baseline tissue sampling by bronchoscopy or effusion collection within 48 h. Patients were subsequently diagnosed to have non-small cell lung carcinoma (NSCLC), other cancers, or not cancer based on histology, cytology, or microbiological testing. For NSCLC patients, the standard of care targeted *EGFR* mutation tissue testing was performed on tumor biopsy samples, where available, using the Roche cobas® EGFR Mutation Test or by Sanger sequencing in a College of American Pathologists (CAP)-accredited clinical laboratory, and results were available as clinical reports. For all patients with blood available, targeted NGS plasma testing was performed in a CAP-accredited clinical laboratory, to detect tumor mutations in cfDNA. Similar targeted NGS testing was also performed in matched tissue samples, for cases with additional tissue available. Basic patient characteristics, namely, age, gender, and confirmed histological diagnosis were recorded as part of the study. This study was approved by the institutional review board of Changi General Hospital and is registered under clinical trial number NCT04254497.

### Plasma and Tissue NGS Genotyping

Peripheral blood was collected in ethylenediaminetetraacetic acid (EDTA) tubes and blood was processed within 24 h of collection to isolate plasma. Circulating nucleic acid was extracted from plasma samples using the QIAamp Circulating Nucleic Acid Kit (Qiagen) and cfDNA was used to perform an NGS assay (LiquidHALLMARK®) in a CAP-accredited clinical laboratory. LiquidHALLMARK® is a clinically validated, ultrasensitive, and amplicon-based assay for the detection of single nucleotide variants (SNVs), insertion-deletion mutations (indels), and copy number alterations among 49 genes (at the time of this study) (Supplementary Table 1) with sensitive detection at variant allele frequencies above 0.1% for SNVs and indels. In this study, clinically actionable mutations were defined as mutations in *EGFR, ERBB2, BRAF, KRAS*, and *MET* (exon 14 skipping and copy number gains) which are therapeutically targetable, guideline-recommended biomarkers or emerging biomarkers for the treatment of metastatic NSCLC (25). Tumor DNA was extracted from remaining available tissue biopsy material using the QIAamp DNA FFPE Tissue Kit (Qiagen) and was also analyzed for panel-wide confirmation and concordance of findings from plasma cfDNA, using the same platform technology as LiquidHALLMARK® (TissueHALLMARK®). The NGS assay did not examine fusions in *ALK, RET*, and *ROS1* at the time of this study.

### Data Analysis

Concordance analysis between routine molecular tissue testing and plasma samples was focused on the presence of mutations in *EGFR* as this is a routine molecular diagnostic test available for patients with NSCLC, ordered by practicing oncologists. Sensitivity and specificity analyses were performed taking tissue *EGFR* test results as standard. Other actionable mutations (non-*EGFR*) detected in *BRAF, KRAS, ERBB2*, and *MET* using plasma NGS panel testing were recorded as additional actionable findings, and any other mutations detected among the 49 genes targeted in the NGS assay were recorded as other genomic findings. The overall rate of detection of mutations in plasma cfDNA NGS was analyzed. For panel-wide testing done on matched plasma and tissue samples (where available), positive and negative predictive agreement analysis was performed for all actionable genomic findings. For NGS, variant allele frequencies (VAFs) were analyzed and are defined as the proportions of variant alleles relative to wild-type alleles. For patients with concurrent plasma and tissue NGS tests, correlation analysis of plasma and tissue variant allele frequencies (AFs) was done using Spearman's rank correlation. Fisher's exact test was used to determine associations between detection of actionable mutations and average coverage, and disease stage for NSCLC. All analyses were performed using RStudio V1.2.5033.

Clinical endpoints included test turnaround time (TAT), measured in days from biopsy sampling to reporting of *EGFR* molecular test results, or from blood sampling to reporting of NGS results.

## Results

### Patient and Sample Characteristics and Test Results Accessibility

A total of 71 patients suspected to have lung cancer, based on their diagnostic scans and symptomatology, were enrolled. Patients were predominantly male (52/71, 73%) and the median age of the patient group was 67 years (range 31–87). Based on histology or cytology specimens, 54 patients (76.1%) were subsequently confirmed to have NSCLC, seven (9.9%) were diagnosed with having other cancers, and the remaining 10 (14.1%) did not have cancer, with diagnosis of tuberculosis, pneumonia or inflammation, or an undetermined noncancer diagnosis (Table 1). Blood samples could be obtained for 99% (70/71) patients before diagnostic biopsy sampling. Among NSCLC cases, blood sample was not available for one patient, resulting in an accessibility rate of 98.4% (53/54) for blood samples. Plasma NGS testing was successful for 100% of blood samples collected (70/70), and among 100% of NSCLC patients with blood samples available (53/53). Volume of plasma available ranged from 0.5 to 9 ml (median, 5.5 ml), and yield of cfDNA was in the range of 10–350 ng per ml plasma (median, 19.24 ng per ml plasma) (Supplementary Figure 1).

**Table 1:** Baseline patient characteristics.

**Characteristics**	**No. (%)**
Total	71 (100)
Sex	
Female	19 (27)
Male	52 (73)
Median age, (range), years	67 (31–87)
Diagnosis ^†^	
NSCLC	54 (76)
adenocarcinoma	37 (52)
squamous cell carcinoma	6 (8.5)
large cell carcinoma	1 (1.4)
NOS	6 (8.5)
with neuroendocrine feature	1 (1.4)
lymphoepithelioma-like carcinoma	3 (4.2)
SCLC	4 (5.6)
Hepatocellular carcinoma	1 (1.4)
High-grade undifferentiated sarcoma	1 (1.4)
Ovarian cancer	1 (1.4)
Tuberculosis	3 (4.2)
Pneumonia	2 (2.8)
Inflammation	3 (4.2)
Undetermined, not cancer	2 (2.8)

† *Diagnosis was determined by histology or cytology, before any tissue or plasma molecular testing, but after blood collection*.

*NSCLC, non-small cell lung carcinoma; SCLC, small cell lung carcinoma; NOS, not otherwise specified*.

**Figure 1:**
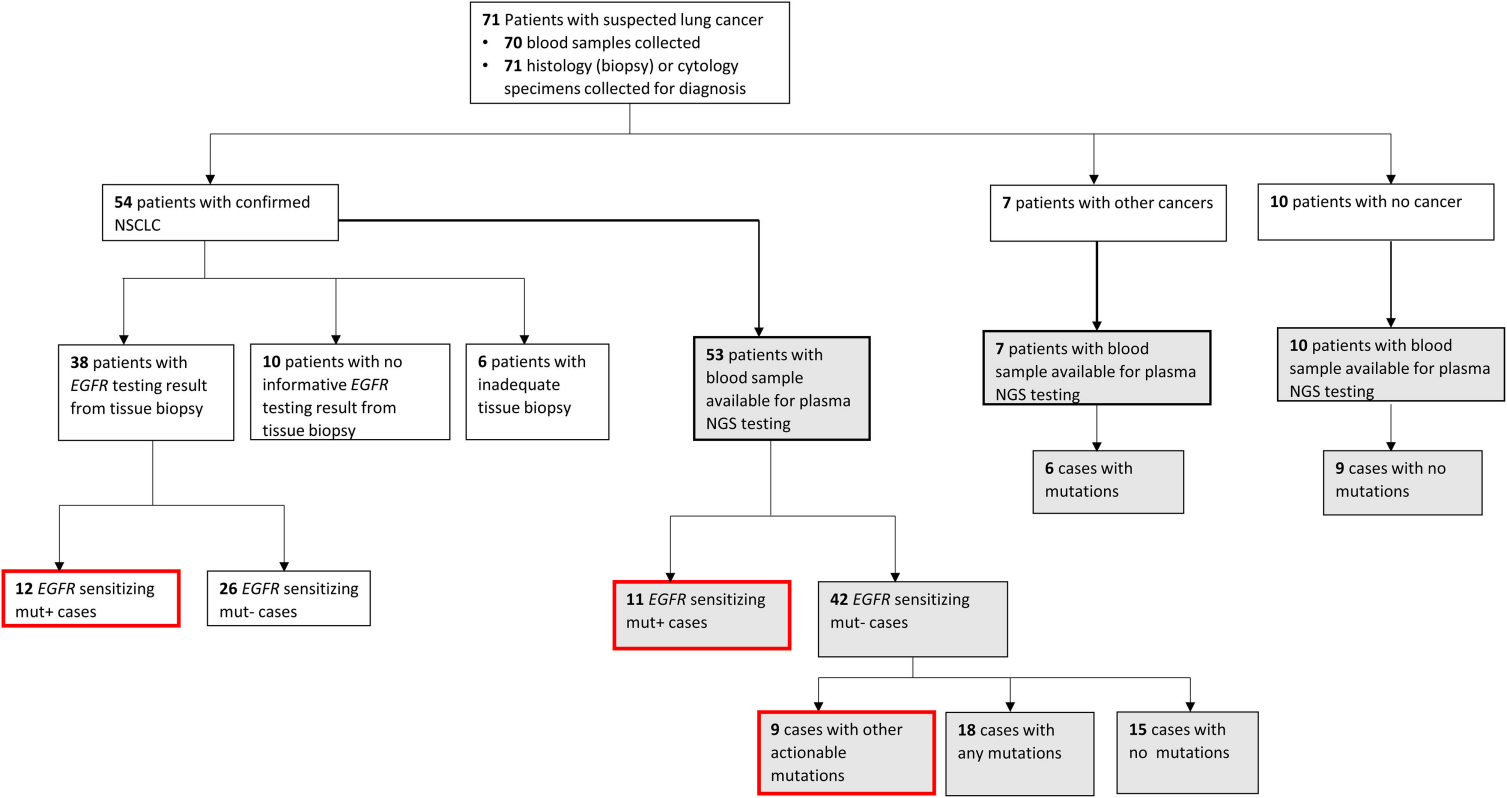
Patient enrollment and testing workflow. Flowchart showing the patient enrolment, diagnosis, sample availability, type of testing conducted, and mutation findings. Gray-filled boxes indicate testing results from plasma next-generation sequencing (NGS) and red-outlined boxes are all clinically actionable findings made in this study for non-small cell lung carcinoma (NSCLC) cases.

Patients with NSCLC (*n* = 54) were eligible for tissue *EGFR* testing, however, tissue *EGFR* test results were available only for 38 NSCLC patients, resulting in a significantly lower tissue results accessibility rate of 70.4% (38/54), with 29.6% of cases (16/54) having no *EGFR* test results from tissue (Supplementary Figure 2). There were two primary reasons for lack of tissue *EGFR* test results for NSCLC patients, namely, failure to obtain adequate biopsy sample due to advanced age of patients or aggressive disease in 37.5% of cases (6/16) and failure to obtain informative *EGFR* test results from collected biopsies for 62.5% of cases (10/16). Patient enrollment, testing workflow and an overview of mutation findings are described in Figure 1.

Among NSCLC cases with successful tissue *EGFR* testing using biopsy, an *EGFR* mutation was found in 31.6% of cases (12/38), whereas an *EGFR* sensitizing mutation was found in 20.7% of cases (11/53) that underwent plasma NGS testing. Among NSCLC cases tested by plasma NGS that were negative for *EGFR* sensitizing mutations, additional actionable findings were made in nine of 42 cases (21.4%), and other genomic findings (any other nonactionable mutations from the 49 gene LiquidHALLMARK® panel) were made in 42.8% (18/42) cases. Of the seven cases subsequently diagnosed by histology or cytology to have other non-NSCLC cancers, six cases had ≥ 1 mutation identified by plasma NGS testing (Supplementary Table 2). In nine of 10 patients with noncancer diagnosis confirmed, no mutations were detected by plasma NGS testing.

### Diagnostic Yield From Tissue Biopsy and Plasma

**Figure 2:**
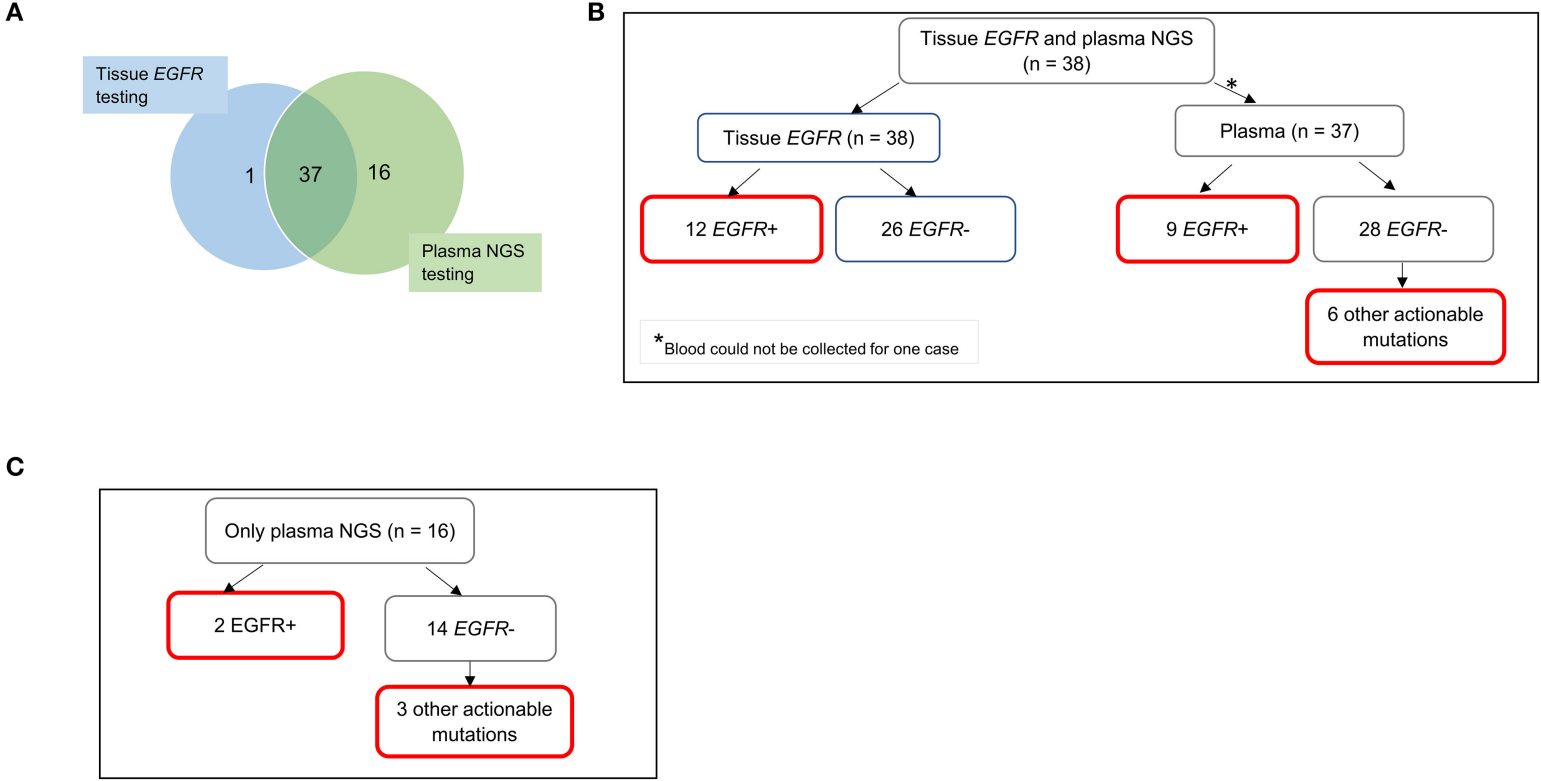
Diagnostic yield from molecular testing of tissue and plasma samples for 54 patients with NSCLC. **(A)** Nearly all 38 patients with informative tissue *EGFR* testing underwent plasma NGS testing (except one). An additional 16 patients had only plasma NGS testing done, due to inadequate tissue biopsy samples for molecular testing or non-informative results from tissue testing. **(B)** Findings of *EGFR* sensitizing mutations and other actionable mutations in cases with both tissue *EGFR* and plasma NGS results. Six other actionable mutations from plasma NGS testing included *MET* exon 14 skipping (*n* = 1), *BRAF* p.V600E (*n* = 1), *BRAF* p.K601E (*n* = 1), *KRAS* p.G12D (*n* = 2), and *EGFR* exon 20 insertion (*n* = 1). **(C)** Clinically actionable findings in cases with only plasma NGS testing. Boxes outlined in red indicate clinically actionable diagnostic yield from all testing modalities. EGFR, epidermal growth factor receptor; NGS, next-generation sequencing.

Diagnostic yield was compared for patients with NSCLC where testing was possible with either standard tissue *EGFR* test with concurrent plasma NGS testing, or with plasma NGS only, as dictated by sample availability (Figure 2A). Of the NSCLC cases with available tissue *EGFR* test results, 31.6% (12/38) were positive for *EGFR* sensitizing mutations while the remaining 68.4% (26/38) had a negative *EGFR* mutation finding. Of these 38 cases, 37 cases were also tested by plasma NGS (blood was not available for one case) with 24.3% (9/37) having a positive result for *EGFR* sensitizing mutation and the remaining 75.7% (28/37) having a negative *EGFR* mutation result (Figure 2B). Specifically, among tissue *EGFR*-negative cases also tested by plasma NGS (*n* = 25), plasma NGS did not identify any further *EGFR* sensitizing mutations (for which FDA-approved therapies are available) but did identify other clinically actionable mutations in six cases, namely, *MET* exon 14 skipping (*n* = 1), *BRAF p*.V600E (*n* = 1), *BRAF p*.K601E (*n* = 1), *KRAS p*,G12D (*n* = 2), and *EGFR* exon 20 insertion (*n* = 1) (Figure 2B).

Importantly, where tissue *EGFR* testing results were lacking and only plasma NGS was performed (*n* = 16), clinically actionable mutations were detectable in five cases, namely, sensitizing *EGFR* mutations p.E746_A750del (*n* = 1) and p.L747_P753delinsS (*n* = 1), *BRAF* p.K601E (*n* = 1), *KRAS* p.G12D (*n* = 1), and *MET* exon 14 skipping (*n* = 1) (Figure 2C).

The additional diagnostic yield from plasma NGS testing for tissue *EGFR*-negative cases is therefore 24% (6/25), for which other actionable mutations were detected. Among NSCLC samples that totally failed to undergo tissue *EGFR* testing (*n* = 16), plasma NGS provided a diagnostic yield of 31.3% (5/16). The total additional diagnostic yield by plasma NGS is therefore 26.8% (11/41).

In this cohort of 54 patients with NSCLC, irrespective of the availability of tissue *EGFR* testing, a plasma NGS test on its own would have provided clinically actionable mutation information in up to 37% of cases (20/54). In contrast, standard tissue *EGFR* testing (with limitations of tissue sampling and quality and breadth of testing), accurately identified only 22.2% (12/54) of cases with clinical actionability based on *EGFR* sensitizing mutations. Performing both tissue and plasma testing resulted in a diagnostic finding in 42.6% (23/54) of NSCLC cases, considering only tissue *EGFR* test and plasma NGS test not including *ALK, RET, ROS1* fusions among actionable targets, which represents a 1.9-fold increase in the number of actionable findings compared to tissue *EGFR* testing alone.

**Figure 3:**
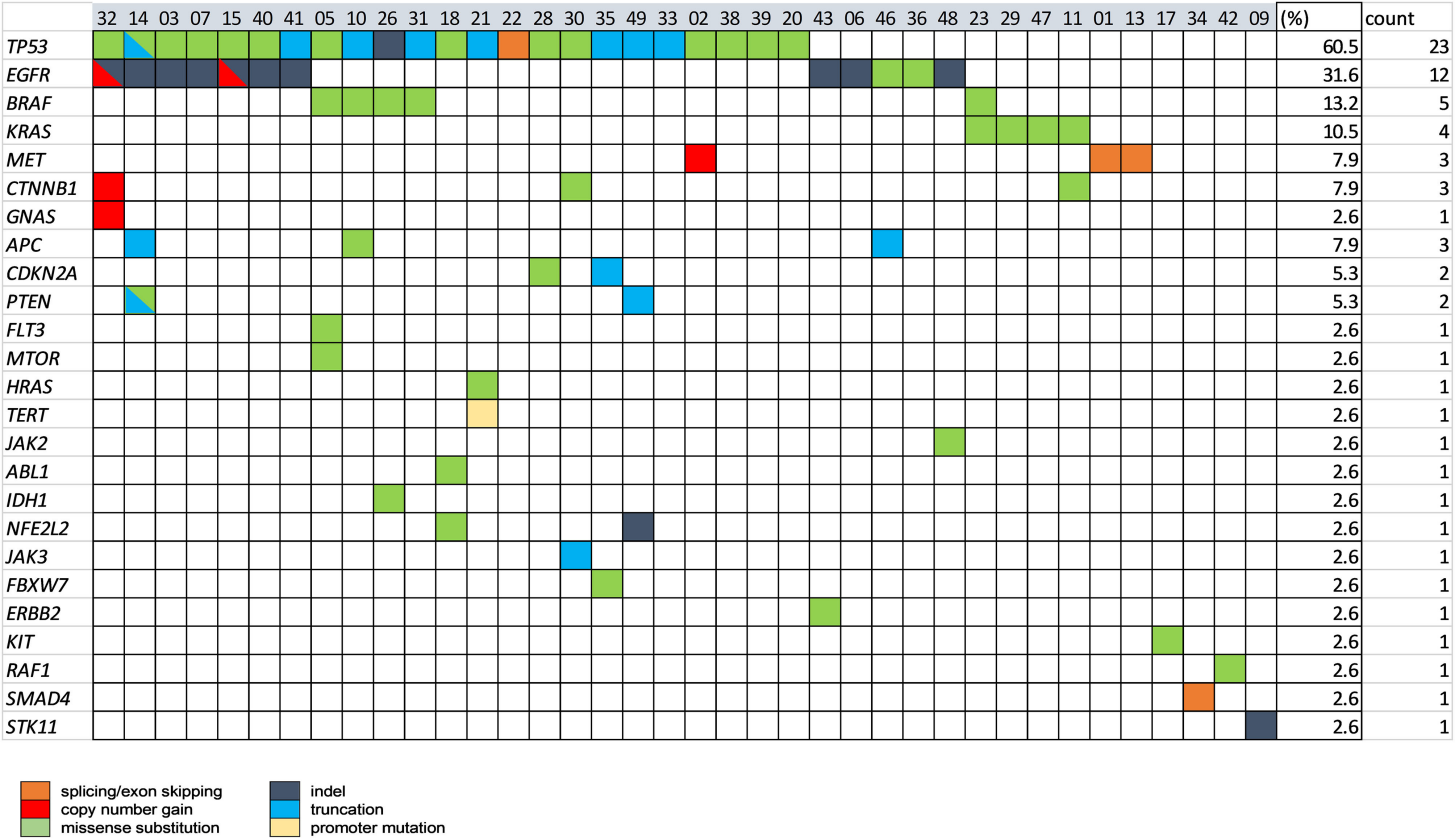
The spectrum of genomic alterations in NSCLC was detected by plasma NGS testing. Baseline plasma samples for 53 NSCLC cases were tested with the 49-gene panel LiquidHALLMARK® assay. Cases with ≥1 alteration are presented (*n* = 38), 15 cases with no alteration detected were excluded from the presentation. Genes with no alteration detected among all cases were also excluded from the presentation. Percentage (%) and number of cases carrying a mutation in each gene are shown in the right-most columns.

The spectrum of all the mutations (actionable and nonactionable) detected by plasma NGS in 53 NSCLC cases is shown in Figure 3. A total of 38 NSCLC cases (76%) had ≥ 1 alteration detectable, of which *TP53* mutations were most prevalent (60.5%), followed by mutations in clinically actionable target genes, *EGFR* (31.6%), *BRAF* (13.2%), *KRAS* (10.5%), and *MET* (7.9%).

### Tissue and Plasma Concordance for EGFR Mutations and Other Variants

To assess the performance of the plasma NGS test relative to the standard tissue *EGFR* testing modality, samples with results from both plasma NGS and tissue tests were compared. Among 12 cases positive for sensitizing *EGFR* mutations by standard *EGFR* tissue testing, nine were found to have the same mutation in plasma cfDNA, for a sensitivity of 75% (9/12) (Table 2). Out of 26 cases negative for sensitizing *EGFR* mutations in tissue, 25 cases were tested by plasma NGS, and concordantly none were found to have any *EGFR* mutations [except for one case with an *EGFR* exon 20 insertion (*EGFR* p.A763_Y764insFQQA)] resulting in a specificity of 100% (25/25). The overall concordance of *EGFR* sensitizing mutations commonly included in the range of PCR-based *EGFR* testing and plasma NGS was 91.9% (34/37). The range of *EGFR* VAFs detected by plasma NGS was 0.057–80.3%, with a median AF of 0.98%, with 7 *EGFR* exon19 deletions and 2 L858R mutations (Table 3). As the detection sensitivity of NGS assays is a function of the depth of coverage achieved, which, in turn, is a function of input DNA amount, we looked at the distribution of depth of coverage across the samples for which *EGFR* mutations were expected to be found in plasma based on tissue results. For three samples in which corresponding *EGFR* mutations were not detected in plasma, the average consensus coverage (X) was 6,524X, 8,068X, and 14,565X, respectively (Table 3), which did not correspond to the lowest coverage among these samples. In fact, two cases with coverage of 4,538X and 4,830X, respectively, had detectable mutations at variant allele frequencies of 0.057 and 9.44% for *EGFR* p.E746_A750del (exon 19 deletion), suggesting a biological (such as low tumor shedding into circulation) rather than a technical reason for discordance. Considering all NSCLC samples tested by plasma NGS (*n* = 53), the median consensus coverage was 8183x. Among samples with coverage lower than the median coverage (*n* = 26), nine samples had no mutations detected by plasma NGS, and among samples with coverage greater than or equal to the median coverage (*n* = 27), six samples had no mutations detected by plasma NGS (*p* = 0.2238, Fisher's exact test), suggesting that coverage was not the main determining factor for detection of variants among these samples (Supplementary Figure 3).

**Table 2:** Concordance analysis of *EGFR* mutation detection by targeted tissue *EGFR* testing and plasma NGS for 37 NSCLC cases.

		**Tissue** ***EGFR*** **testing (Sanger or targeted PCR)**			
		**Positive**	**Negative**	**Total**	
Plasma NGS	Positive	9	0^†^	9	Sensitivity: 75% (95% CI: 42.8–94.5%) Specificity: 100% (95% CI: 86.3% to 100.0%) Accuracy: 91.9% (95% CI: 78.1% to 98.3%)
	Negative	3	25	28	

*Sensitivity of plasma NGS for EGFR detection relative to tissue EGFR testing was 75% (9/12) and specificity was 100% (25/25), for an overall concordance of 91.9% (34/37)*.

† *One case in plasma NGS was found to have EGFR p.A763_Y764insFQQA, an exon 20 insertion mutation, which is not part of the assay used in routine PCR-based tissue EGFR testing, and is not included in the count*.

**Table 3:** Depth of coverage by plasma NGS and detection of *EGFR* mutation and mutation allele frequency (AF %).

	**Tissue** ***EGFR*** **test**		**Plasma NGS test**		
**Case**	***EGFR*** **Mutation**	**Method**	**Average consensus coverage (X)**	***EGFR*** **Mutation (HGVSp)**	**AF (%)**
1	Exon19del	Sanger	9496.7	p.E746_A750del	0.33
2	Exon19del	Roche PCR	4830.58	p.E746_A750del	9.44
3	Exon19del	Roche PCR	14854.77	p.E746_A750del	7.15
4	Exon19del	Sanger	11864.58	p.E746_S752delinsV	50.7
5	Exon19del	Roche PCR	6524.41	-	ND
6	p.E746_T751delinsA	Sanger	12254.13	p.E746_T751delinsA	80.3
7	p.L858R	Roche PCR	13842.24	p.L858R	1.51
8	Exon19del	Sanger	8068.15	-	ND
9	p.L858R	Sanger	14565.16	-	ND
	p.E709K			-	ND
10	p.L747_A750delinsP	Sanger	7420.09	p.L747_A750delinsP	0.98
11	p.L858R	Roche PCR	10417.81	p.L858R	0.86
	p.S768I			p.S768I	0.25
12	Exon19del unspecified	Roche PCR	5722.85	p.E746_A750del	0.73

*ND, not detected*.

Beyond *EGFR*, panel-wide concordance of mutation findings in tissue biopsy samples and plasma was studied by performing tissue NGS using the same panel (TissueHALLMARK®) on a subset of samples for which tissue samples from the original biopsy were available. A total of 24 patients with NSCLC had both the plasma NGS and tissue NGS results available, of which 14 (58.3%) cases had a therapeutically relevant target detected, either by plasma or tissue NGS or by both methods. The positive predictive agreement (PPA) between plasma and tissue NGS was 75.0% and the negative predictive agreement (NPA) was 83.3%, for an overall predictive agreement (OPA) of 79.2% (Table 4). There was a correlation between the plasma and tissue mutation AF among actionable mutations detected (ρ = 0.5503, *p* = 0.0221) (Supplementary Figure 4). It was observed that for cases in which tissue mutation was not detected in plasma, the AF was low in the tissue sample, below 10% AF. Conversely, two mutations identified only in plasma were characterized by very low AF −0.04 and 0.3% (Supplementary Figure 4). To account for the discordant mutations, the extent of clinical disease was examined by comparing tumor stage information, which was available only for 49% of NSCLC cases in this study that underwent plasma NGS (26/53). Actionable mutations were detected in plasma for 0% (0/6) cases with disease stage 2B-3B, including one tissue-discordant *EGFR* sensitizing mutation (Table 5). In contrast, for cases with disease stage 4 or 4B (*n* = 20), an actionable mutation was detected in 45% (9/20) cases, including five tissue concordant *EGFR* sensitizing mutations (Stage 2B-3B vs. Stage 4-4B: Fisher's exact test, *p* = 0.0632) (Tables 5, 6).

**Table 4:** Panel-wide concordance of actionable mutations in 24 NSCLC cases that underwent both the tissue and plasma NGS testing.

		**Tissue NGS**			
		**Positive**	**Negative**	**Total**	
Plasma NGS	Positive	9	2	11	PPA: 75.0% (95% CI: 42.8 to 94.5%) NPA: 83.3% (95% CI: 51.6 to 97.9%) OPA: 79.2% (95% CI: 57.9 to 92.9%)
	Negative	3	10	13	

*PPA, positive percent agreement; NPA, negative percent agreement; OPA, overall percent agreement*.

**Table 5:** Cancer stage-dependence of detection of actionable mutations in plasma.

		**Stage**	
		**2B-3B**	**4-4B**
Actionable mutation	Detected	0	9
	Not detected	6	11

*Fisher's exact test, p-value = 0.0632*.

**Table 6:** Clinically actionable mutations detected in stage 4-4B cases and their concordance of detection with tissue *EGFR* tests.

**Stage**	**Mutation (HGVSp)**	**AF (%)**	**Concordant with tissue *EGFR***
4B	*EGFR* p.E746_A750del	7.15	Yes
4	*EGFR* p.E746_A750del	0.73	Yes
4	*KRAS* p.G12D	12.36	NA
4	*EGFR* p.L747_A750delinsP	0.98	Yes
4	*BRAF* p.K601E	12.45	NA
4	*KRAS* p.G12D	27.17	NA
4	*EGFR* p.E746_A750del	0.33	Yes
4	*EGFR* p.E746_S752delinsV	50.7	Yes
4	*KRAS* p.G12D	3.32	NA

*NA, not applicable as not tested by tissue EGFR*.

### Plasma NGS for Non-NSCLC Cancers and Noncancer Samples

As described in Figure 1, plasma samples from patients initially suspected to have lung cancer, but later confirmed to have either other cancers (*n* = 7) or a noncancer diagnosis (*n* = 10), were also tested by NGS. The specificity of detection of cancer-specific mutations by plasma NGS was demonstrated by the detection of a mutation in 85% (6/7) of other cancer cases, including pathogenic *TP53* mutations in 71% (5/7) of cases (Supplementary Table 2). Importantly, among plasma from 10 noncancer cases, only one case harbored an *ALK* frameshift mutation of uncertain significance, which was also present in a pleural effusion sample from the same case (data not shown). This demonstrates that mutation detection by plasma NGS is reliable and specific to the presence of cancer.

### Plasma NGS TAT

Plasma NGS was successfully performed in 53 patients with NSCLC and 17 patients with non-NSCLC with suspected lung cancer with an average TAT of 10 days from the time of blood draw to the time of receipt of the report. In contrast, the average TAT for tissue NGS for 38 patients with standard *EGFR* testing with tissue was 29.9 days (*p* < 0.05), with the longest duration between biopsy collection and receipt being 48 days.

## Discussion

In this single-center prospective study, we assessed the clinical utility of adding plasma NGS testing to the diagnostic workflow for suspected lung cancer and molecular testing workflow for diagnosed NSCLC. This approach may be labeled as “plasma-first” for cases with no tissue sample available for testing, or complementary where both tumor and plasma sample may be tested for comprehensive target coverage, or where there is uncertainty about the adequacy of a tissue sample for molecular testing (19). Plasma NGS demonstrated significantly higher sample accessibility levels, lower average reporting time, and matched specificity and accuracy when compared to standard tissue *EGFR* testing. Importantly, a range of additional actionable mutations from guideline-recommended biomarkers was found by plasma NGS, potentially enabling an appropriate targeted treatment option, even in the absence of a tissue test result.

The invasive nature of tissue biopsy makes routine diagnostic EGFR profiling unfeasible for patients with late-stage NSCLC and those of advanced age. In this study, only 70.3% (38/54) of diagnosed patients with NSCLC had informative results from tissue *EGFR* testing. On the other hand, the blood sample was collected for 99% of all the patients recruited in this prospective study (70/71), including 98.4% (53/54) of patients with NSCLC. We show the clinical value of a plasma NGS test was an average 26.8% additional diagnostic yield over tissue *EGFR* testing, from the combined contribution of (1) additional actionable mutations in six of 25 tissue *EGFR*-negative cases and (2) detection of five actionable mutations, including 2 *EGFR* sensitizing mutations, in 16 cases that had no results from tissue *EGFR* testing.

Among all the patients with NSCLC, adding plasma NGS to tissue *EGFR* testing resulted in the detection of a therapeutically actionable mutation in 43.6% (23/54) cases, whereas if only tissue *EGFR* testing had been done, only 22.2% (12/54) cases would have had a clinical actionable finding. This represents a 1.9-fold increase in the number of actionable mutations detected in this study by adding plasma NGS testing, including two *EGFR* mutations in two cases that failed standard tissue *EGFR* testing. This is consistent with past studies in larger real-world NSCLC cohorts, where the addition of comprehensive liquid biopsy to targeted tissue testing increased the number of targetable mutations up to as much as 65% (17, 26). This makes plasma NGS an especially important diagnostic tool when tissue biopsy is scant or not available.

The routine implementation of a complementary plasma or even “plasma-first” testing approach in healthcare settings significantly reduces reporting time and can enable patients to begin targeted therapy earlier. Tissue *EGFR* results took an average of 29.9 days to report, while plasma NGS took an average of 10 days to report. In this study, for 37% (20/54) of NSCLC cases, a treatment decision could have been made as soon as the plasma NGS results became available. This trend of a lower turnaround time with plasma NGS tests has been widely supported by other studies (27, 28). The length of time between the scheduling of the tissue biopsy and the procedure itself can vary widely and can add many weeks to an already long wait for a diagnosis. In contrast, in-clinic, same-day blood collection for plasma NGS can be quickly and conveniently performed.

The high specificity of diagnostics is key to ensuring that false-positive findings do not result in incorrect treatment, which can be harmful to patients and increases the financial burden of healthcare. The specificity of plasma NGS compared with PCR-based tissue *EGFR* testing in this study was 100%. This finding provides supporting evidence that positive identification of an actionable mutation by plasma NGS is sufficient evidence to initiate targeted treatments without needing additional confirmation from tissue testing (26), reducing the duration from clinical consultation to the start of the treatment program. In this study, tissue *EGFR* results would have yielded additional findings in 5.56% (3/54) of cases, for which plasma NGS did not find the *EGFR* mutation present in the tumor, supporting the complementary plasma testing would be the most informative approach for the NSCLC patient population.

Plasma NGS reported a sensitivity of 75% when compared to routine Sanger or targeted PCR, which suggests that negative results require further investigation to rule out the possibility of false negatives. Levels of circulating tumor DNA (ctDNA) are highly varied between patients, likely because ctDNA levels can vary based on the rate of turnover, perfusion, and vascularization of the tumor, and are influenced by the cancer stage (29). In this study, a disease stage-dependence was observed for both *EGFR* mutation concordance and detection rate of any actionable mutation, with 45% (9/20) of cases with stage 4 or 4B vs. 0% (0/6) of cases with stage 2B-3B having an actionable mutation detected. Further, there was a correlation between the tumor AF and plasma AF of mutations for cases where both plasma and tissue NGS were performed, suggesting ctDNA burden is a function of the actual tumor size and spread. This is in alignment with another study where patients with liver metastases had higher plasma-tissue concordance for actionable mutations compared to those with M1a disease (17), and with a study in which patients with intrathoracic metastases alone were less likely to have detectable ctDNA (30). It has been suggested that the disease stage could serve as a decision metric to decide the order in which plasma or tissue testing is requested, to maximize detection of actionable mutations detection without unnecessarily prolonging the time to result.

As an attestation of the specificity and broad applicability of plasma NGS for cancer diagnostics, we also show that among other suspected lung cancer patients eventually diagnosed to not have cancer, only one case (out of 10) had a detectable mutation (of uncertain significance), while six of seven cases diagnosed as having other non-NSCLC cancers had *TP53* mutations or other cancer-related mutations detected. Based on these results, a role for plasma NGS testing in preliminary cancer diagnosis could be envisioned.

This study has limitations in that the standard diagnostic test comparison was limited to *EGFR* mutations, and actionable fusions in *ALK, ROS1, RET*, and *NTRK* were not considered as they were not measured at the time of this study. Including actionable fusions in concurrent tissue and plasma, NGS tests will likely result in a similar fractional increase in actionability. Another limitation was that disease stage information was only available for a subset of patients, limiting the stage-specific analysis for plasma-tissue concordance. Furthermore, the study was conducted in a small cohort of prospectively recruited patients and no information on treatment decisions and clinical outcomes was recorded, which would have enabled the real-world clinical utility of the complementary or “plasma-first” approach to be quantified in a prospective setting. Finally, longitudinal monitoring of the efficacy of plasma NGS on this patient cohort was not captured in this study. However, the non-invasive nature and sensitive detection ability of plasma NGS make it a suitable tool for the determination of resistance mutations earlier and with greater accessibility than would be possible with an initial biopsy or rebiopsy.

This study demonstrates that integrating plasma NGS with tissue testing increases actionable yield over conventional diagnostic approaches for NSCLC by allowing more patients to achieve comprehensive biomarker profiling. Plasma NGS allows for quick and non-invasive molecular profiling that can rapidly guide treatment decisions and complement routine tissue testing or tissue NGS or could be a viable first-line alternative when tissue biopsy is not feasible.

## Data Availability Statement

The original contributions presented in the study are included in the article/Supplementary Material, further inquiries can be directed to the corresponding author.

## Ethics Statement

The studies involving human participants were reviewed and approved by Institutional Review Board of Changi General Hospital. Study is registered under clinical trial number NCT04254497. The patients/participants provided their written informed consent to participate in this study.

## Author Contributions

YC, M-HT, and JM conceived and planned the experiments. JM, M-HT, and AT contributed to the samples preparation. YC, JP, and KN carried out the experiments and preliminary analysis. YC, JP, KN, JM, and RG analyzed and interpreted the results. YC wrote the manuscript with support from RG and JM. All authors provided critical feedback and helped shape the analysis and manuscript and contributed to the article and approved the submitted version.

## Funding

The study was funded by the Agency for Science, Technology and Research Biomedical Research Council-Economic Development Board (A^*^STAR BMRC-EDB) Industrial Alignment Fund (IAF) (Project No. IAF111221).

## Conflict of Interest

YC, M-HT, JP, and KN are employees of Lucence Diagnostics Pte Ltd.

The remaining authors declare that the research was conducted in the absence of any commercial or financial relationships that could be construed as a potential conflict of interest.

## Publisher's Note

All claims expressed in this article are solely those of the authors and do not necessarily represent those of their affiliated organizations, or those of the publisher, the editors and the reviewers. Any product that may be evaluated in this article, or claim that may be made by its manufacturer, is not guaranteed or endorsed by the publisher.

## References

[1] Molina JR Yang P Cassivi SD Schild SE Adjei AA. Non-small cell lung cancer: epidemiology, risk factors, treatment, and survivorship. Mayo Clin Proc. (2008) 83:584–94. 10.1016/S0025-6196(11)60735-0 18452692 PMC2718421

[2] SEER Cancer Statistics Review (CSR) 1975-2015 (2018). Available online at: https://seer.cancer.gov/archive/csr/1975_2015/

[3] Lindeman NI Cagle PT Aisner DL Arcila ME Beasley MB Bernicker EH. Updated molecular testing guideline for the selection of lung cancer patients for treatment with targeted tyrosine kinase inhibitors: guideline from the college of american pathologists, the international association for the study of lung cancer, and the association for molecular pathology. Arch Pathol Lab Med. (2018) 142:321–46. 10.5858/arpa.2017-0388-CP 29355391

[4] Hanna N Johnson D Temin S Baker S Brahmer J Ellis PM. Systemic Therapy for stage IV non–small-cell lung cancer: American society of clinical oncology clinical practice guideline update. JCO. (2017) 35:3484–515. 10.1200/JCO.2017.74.6065 28806116

[5] Chen R Manochakian R James L Azzouqa A-G Shi H Zhang Y. Emerging therapeutic agents for advanced non-small cell lung cancer. J Hematol Oncol. (2020) 13:58. 10.1186/s13045-020-00881-7 32448366 PMC7245927

[6] Alexander M Kim SY Cheng H. Update 2020: management of non-small cell lung cancer. Lung. (2020) 198:897–907. 10.1007/s00408-020-00407-5 33175991 PMC7656891

[7] Skoulidis F Li BT Dy GK Price TJ Falchook GS Wolf J. Sotorasib for Lung Cancers with KRAS pG12C Mutation. N Engl J Med. (2021) 384:2371–81. 10.1056/NEJMoa2103695 34096690 PMC9116274

[8] National Comprehensive Cancer Network (NCCN) NCCN Clinical Practice Guidelines in Oncology: Non-Small Cell Lung Cancer. Version 5.2021. Available online at: https://www.nccn.org/professionals/physician_gls/pdf/nscl.pdf

[9] Gutierrez ME Choi K Lanman RB Licitra EJ Skrzypczak SM Pe Benito R. Genomic profiling of advanced non–small cell lung cancer in community settings: gaps and opportunities. Clin Lung Cancer. (2017) 18:651–9. 10.1016/j.cllc.2017.04.004 28479369

[10] Lim C Tsao MS Le LW Shepherd FA Feld R Burkes RL. Biomarker testing and time to treatment decision in patients with advanced nonsmall-cell lung cancer†. Ann Oncol. (2015) 26:1415–21. 10.1093/annonc/mdv208 25922063

[11] Makarem M Leighl NB. Molecular testing for lung adenocarcinoma: is it time to adopt a “plasma-first” approach? Cancer. (2020) 126:3176–80. 10.1002/cncr.32875 32365225

[12] Russano M Napolitano A Ribelli G Iuliani M Simonetti S Citarella F. Liquid biopsy and tumor heterogeneity in metastatic solid tumors: the potentiality of blood samples. J Exp Clin Cancer Res. (2020) 39:95. 10.1186/s13046-020-01601-2 32460897 PMC7254767

[13] Boskovic T Stanic J Pena-Karan S Zarogoulidis P Drevelegas K Katsikogiannis N. Pneumothorax after transthoracic needle biopsy of lung lesions under CT guidance. J Thorac Dis. (2014) 6 Suppl 1:S99–107. 10.3978/j.issn.2072-1439.2013.12.08 24672704 PMC3966161

[14] Cescon DW Bratman SV Chan SM Siu LL. Circulating tumor DNA and liquid biopsy in oncology. Nature Cancer. (2020) 1:276–90. 10.1038/s43018-020-0043-5 35122035

[15] Odegaard JI Vincent JJ Mortimer S Vowles JV Ulrich BC Banks KC. Validation of a plasma-based comprehensive cancer genotyping assay utilizing orthogonal tissue- and plasma-based methodologies. Clin Cancer Res. (2018) 24:3539. 10.1158/1078-0432.CCR-17-3831 29691297

[16] Bettegowda C Sausen M Leary RJ Kinde I Wang Y Agrawal N. Detection of circulating tumor DNA in early- and late-stage human malignancies. Sci Transl Med. (2014) 6:224ra24. 10.1158/1538-7445.AM2014-5606 24553385 PMC4017867

[17] Aggarwal C Thompson JC Black TA Katz SI Fan R Yee SS. Clinical Implications of plasma-based genotyping with the delivery of personalized therapy in metastatic non–small cell lung cancer. JAMA Oncology. (2019) 5:173–80. 10.1001/jamaoncol.2018.4305 30325992 PMC6396811

[18] Wang N Zhang X Wang F Zhang M Sun B Yin W. The Diagnostic accuracy of liquid biopsy in EGFR-Mutated NSCLC: a systematic review and meta-analysis of 40 studies. SLAS Technol. (2021) 26:42–54. 10.1177/2472630320939565 32659150

[19] Rolfo C Mack P Scagliotti GV Aggarwal C Arcila ME Barlesi F. Liquid biopsy for advanced non-small cell lung cancer: a consensus statement from the international association for the study of lung cancer (IASLC). J Thorac Oncol. (2021) 16:1647–62. 10.1016/j.jtho.2021.06.017 34246791

[20] Thompson JC Yee SS Troxel AB Savitch SL Fan R Balli D. Detection of Therapeutically Targetable Driver and Resistance Mutations in Lung Cancer Patients by Next-Generation Sequencing of Cell-Free Circulating Tumor DNA. Clin Cancer Res. (2016) 22:5772. 10.1158/1078-0432.CCR-16-1231 27601595 PMC5448134

[21] Schwaederlé MC Patel SP Husain H Ikeda M Lanman RB Banks KC. Utility of genomic assessment of blood-derived circulating tumor DNA (ctDNA) in patients with advanced lung adenocarcinoma. Clin Cancer Res. (2017) 23:5101. 10.1158/1078-0432.CCR-16-2497 28539465 PMC5581668

[22] Hsu W-H Yang JC-H Mok TS Loong HH. Overview of current systemic management of EGFR-mutant NSCLC. Ann Oncol. (2018) 29:i3–9. 10.1093/annonc/mdx702 29462253

[23] Kumari N Singh S Haloi D Mishra SK Krishnani N Nath A. Epidermal growth factor receptor mutation frequency in squamous cell carcinoma and its diagnostic performance in cytological samples: a molecular and immunohistochemical study. World J Oncol. (2019) 10:142–50. 10.14740/wjon1204 31312281 PMC6615915

[24] Liam C-K Stone E Andarini S Liam Y-S Lam DC-L Lee P. Molecular testing of metastatic non-small cell lung cancer in the Asia-Pacific region. Respirology. (2020) 25:685–7. 10.1111/resp.13833 32363718

[25] Ettinger DS Wood DE Aisner DL Akerley W Bauman JR Bharat A. NCCN guidelines insights: non–small cell lung cancer, version 2.2021: featured updates to the NCCN guidelines. J Natl Compr Canc Netw. (2021) 19:254–66. 10.6004/jnccn.2021.0013 33668021

[26] Mack PC Banks KC Espenschied CR Burich RA Zill OA Lee CE. Spectrum of driver mutations and clinical impact of circulating tumor DNA analysis in non–small cell lung cancer: analysis of over 8000 cases. Cancer. (2020) 126:3219–28. 10.1002/cncr.32876 32365229 PMC7383626

[27] Leighl NB Page RD Raymond VM Daniel DB Divers SG Reckamp KL. Clinical utility of comprehensive cell-free DNA analysis to identify genomic biomarkers in patients with newly diagnosed metastatic non–small cell lung cancer. Clin Cancer Res. (2019) 25:4691. 10.1158/1078-0432.CCR-19-0624 30988079

[28] Sabari JK Offin M Stephens D Ni A Lee A Pavlakis N. A prospective study of circulating tumor DNA to guide matched targeted therapy in lung cancers. JNCI. (2019) 111:575–83. 10.1093/jnci/djy156 30496436 PMC6579739

[29] Bronkhorst AJ Ungerer V Holdenrieder S. The emerging role of cell-free DNA as a molecular marker for cancer management. Biomol Detect Quantif. (2019) 17:100087–100087. 10.1016/j.bdq.2019.100087 30923679 PMC6425120

[30] Karlovich C Goldman JW Sun J-M Mann E Sequist LV Konopa K. Assessment of EGFR mutation status in matched plasma and tumor tissue of NSCLC patients from a phase I study of rociletinib (CO-1686). Clin Cancer Res. (2016) 22:2386. 10.1158/1078-0432.CCR-15-1260 26747242 PMC6886231

