# Supplementary material for: Complementing Tissue Testing With Plasma Mutation Profiling Improves Therapeutic Decision-Making for Patients With Lung Cancer
Source: Front Med (Lausanne). 2022 Feb 11;9:758464. doi: 10.3389/fmed.2022.758464 (PMC8873935; doi:10.3389/fmed.2022.758464)
Supplement: Supplementary file 1 [file Data_Sheet_1.pdf]

SUPPLEMENTARY DATA

Complementing Tissue Testing with Plasma Mutation Profiling Improves Therapeutic Decision Making for Lung Cancer Patients

Yukti Choudhury[1], Min-Han Tan[2], Jun Li Shi[3], Augustine Tee[4], Kao Chin Ngeow[1], Jonathan Poh[1], Ruth Rosalyn Goh[1,5], Jamie Mong[3,‡]

[1] Lucence Diagnostics Pte. Ltd., Singapore
[2] Lucence Health Inc, Palo Alto, CA, United States
[3] Institute of Bioengineering and Bioimaging, Singapore
[4] Department of Respiratory and Critical Care Medicine, Changi General Hospital, Singapore
[5] Faculty of Medicine, Imperial College London

SUPPLEMENTARY FIGURES AND TABLES

Table S1. Composition of gene panel used for plasma NGS test (LiquidHALLMARK®). The same gene panel was used for tissue NGS (TissueHALLMARK®) for panel-wide comparison of mutations. *Genes for which copy number alterations can be calculated in both plasma and tissue tests.

	LiquidHALLMARK® Panel (49 Genes)									
SNVs, indels	*ABL1*	*AKT1*	*ALK**	*APC*	*AR**	*ATM*	*BRAF*	*CCND1**	*CDH1*	*CDKN2A**
	CTNNB1	*EGFR**	*ERBB2**	*ESR1*	*FBXW7*	*FGFR2*	*FGFR3*	*FLT3*	*GATA3*	*GNA11*
	GNAQ	*GNAS*	*HNF1A*	*HRAS*	*IDH1*	*IDH2*	*JAK1*	*JAK2*	*JAK3*	*KIT*
	KRAS	*MAPK1*	*MAP2K1*	*MED12*	*MET**	*MTOR*	*MYC**	*NFE2L2*	*NOTCH1*	*NRAS*
	PDGFRA	*PIK3CA**	*PTEN**	*RAF1*	*SMAD4*	*STK11*	*TERT*	*TP53**	*VHL*	

Figure S1. Distribution of (A) volumes of plasma (ml) and (B) yields of cfDNA per ml of plasma for 70 patients. Median and interquartile ranges are shown with red lines. **(C)** Yield of cfDNA per ml distribution by volume, dotted red line shows median cfDNA per ml (ng) amount = 19.24 ng.

(A)

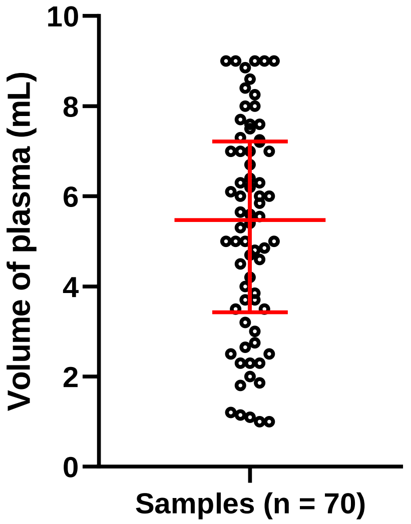

(B)

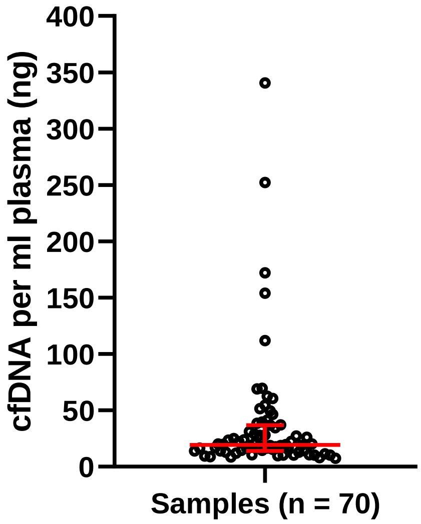

(C)

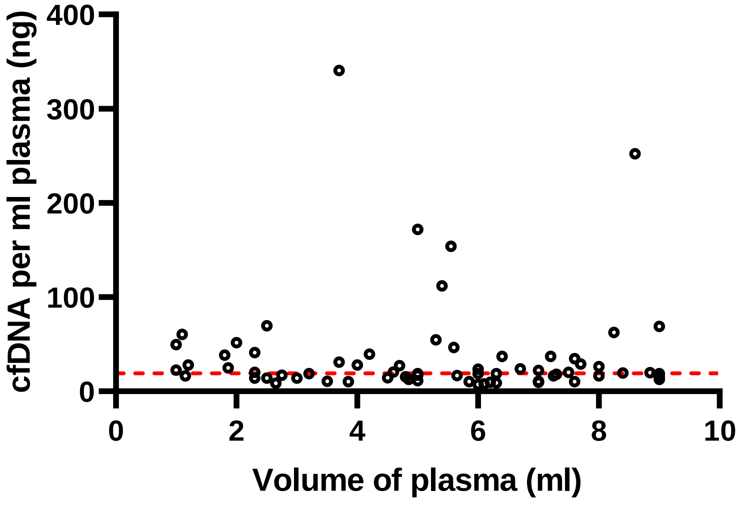

Figure S2. Availability of *EGFR* test results from tissue biopsy samples among 54 NSCLC patients from a total 71 patients suspected to have lung cancer.

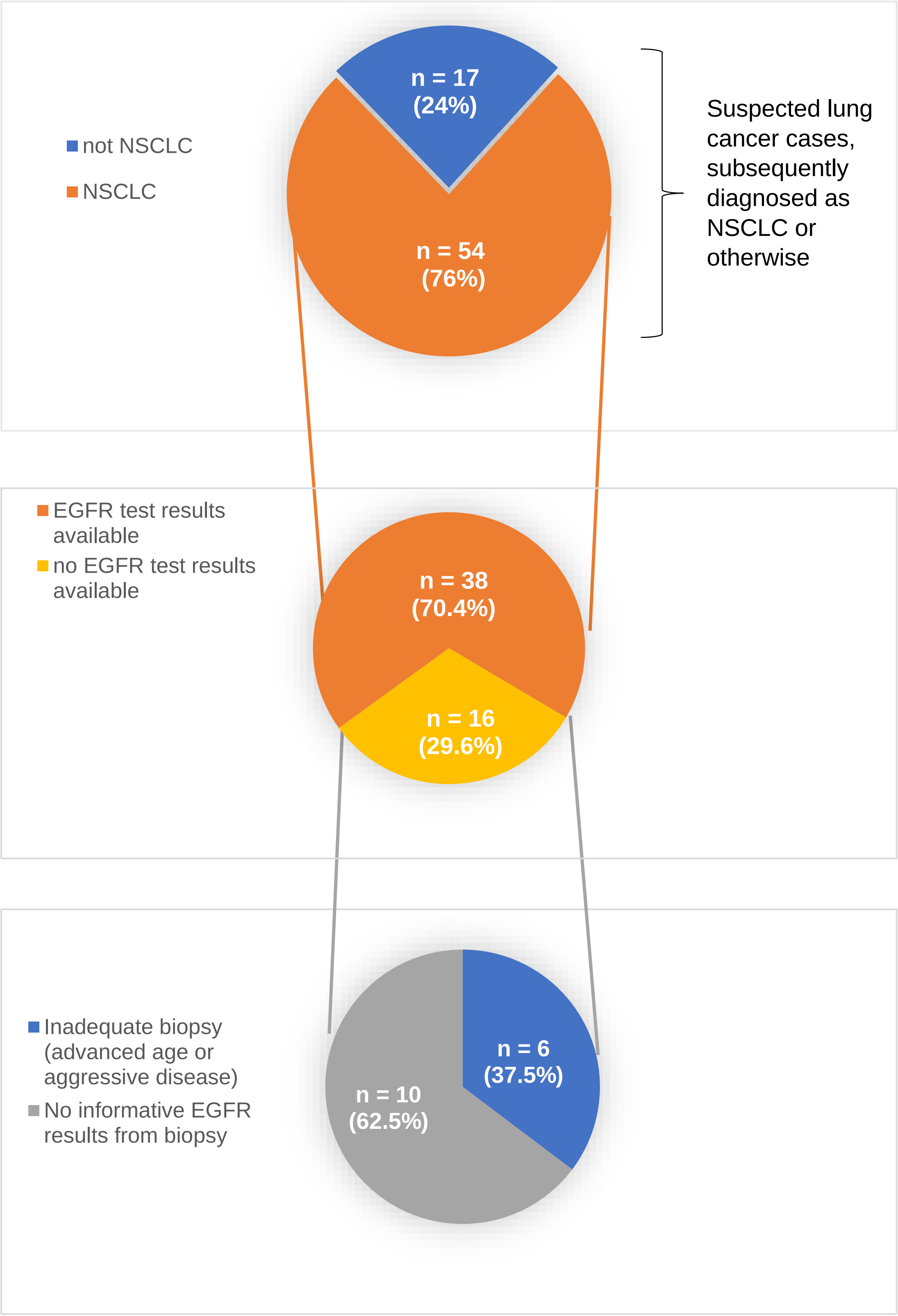

Figure S3. Distribution of average consensus coverage for plasma NGS across 53 NSCLC samples. Average consensus coverage colored by detection of any mutations by plasma NGS, and samples in which *EGFR* mutation was not concordantly detected in plasma NGS. Median (8183x) and interquartile ranges for coverage are shown.

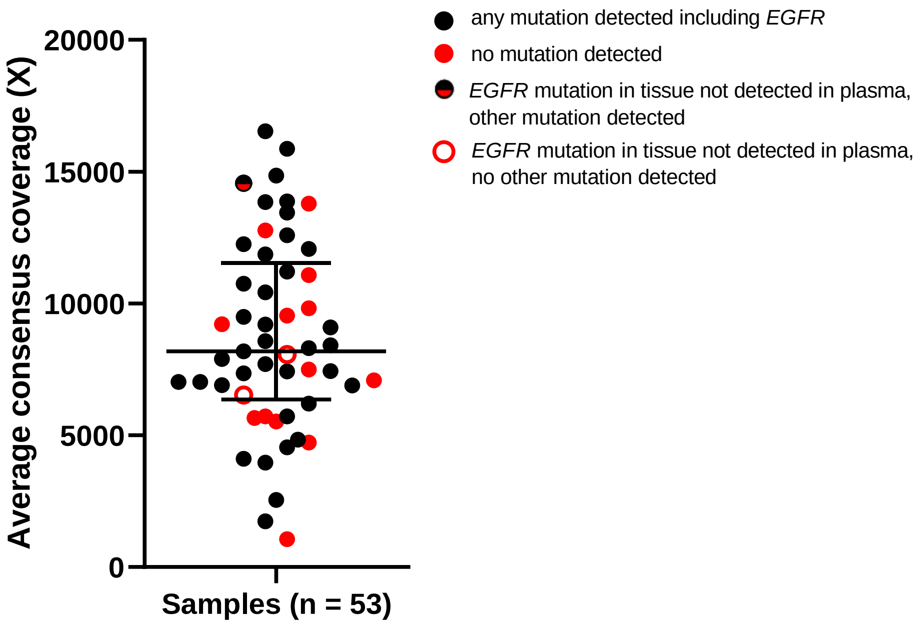

Figure S4. Allele frequencies (AF) of therapeutically actionable mutations detected by plasma NGS and tissue NGS are correlated. For the same patient's blood and tissue AFs of mutations detected are correlated (ρ = 0.5503, p-value = 0.0221). Red circles indicate cases where mutation was only detected in tissue, while green circles indicate mutations found only in plasma. All discordances were characterized by low detectable AFs, below 10% AF for mutations found only in tissue, and below 1% AF for plasma-only mutations.

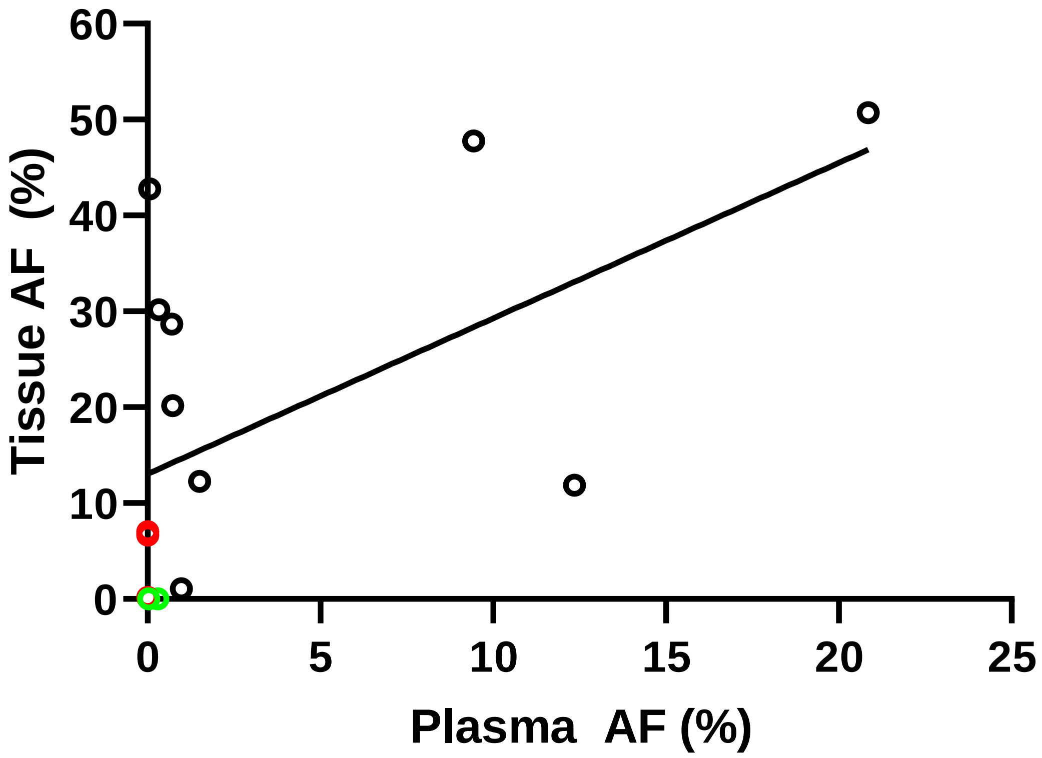

Table S2. Frequent detection of cancer-specific mutations for non-NSCLC cancer patients by plasma NGS. SCLC = small cell lung carcinoma; HCC = hepatocellular carcinoma.

Case	Diagnosis	Mutation (HGVSp)	AF (%)
1	SCLC	*TP53* p.Pro278Arg	27.11
2	SCLC	*TP53* p.Tyr163Cys	12.78
3	HCC	*TP53* p.Arg249Ser<br>*CTNNB1* p.Asp32Ala	1.4<br>0.67
4	High grade undifferentiated sarcoma	-	
5	Metastatic Ovarian Cancer	*STK11* p.Pro315Leu	0.38
6	SCLC	*TP53* p.Arg249GlyfsTer96	3.82
7	SCLC	*TP53* p.Gly154Val	27.6
